# Preventive effect and mechanism of Tibetan tea extract on thrombosis in arachidonic acid-induced zebrafish determined via RNA-seq transcriptome profiles

**DOI:** 10.1371/journal.pone.0285216

**Published:** 2023-05-19

**Authors:** Ning Wang, Chaohua Lan, Huiqiang Lu, Linman Li, Dalong Liao, Kewei Xu, Haiyan Sun, Yongqing Tang, Yumeng Wang, Jie Mei, Mengting Wei, Tao Wu, Hui Zhu

**Affiliations:** 1 College of Bioengineering, Sichuan University of Science and Engineering, Zigong, China; 2 Luzhou Laojiao Co. Ltd, Luzhou, PR China; 3 College of Horticulture, Hunan Agricultural University, Changsha, China; 4 Chengdu Chongqing Shuangcheng Economic Circle (Luzhou) Advanced Technology Research Institute, Luzhou, China; 5 Center for Drug Screening and Research, School of Geography and Environmental Engineering, Gannan Normal University, Ganzhou, China; 6 Bristol Myers Squibb, Princeton, NJ, United States of America; 7 Sichuan Jixiang Tea Co., Ltd., Ya’an, China; 8 School of Food and Biological Engineering, Xihua University, Chengdu, China; Zagazig University, EGYPT

## Abstract

Thrombosis is a key pathological event in cardiovascular diseases and is also the most important targeting process for their clinical management. In this study, arachidonic acid (AA) was used to induce thrombus formation in zebrafish larvae. Blood flow, red blood cell (RBCs) aggregation and cellular oxidative stress were measured to evaluate the antithrombotic effect of Tibetan tea (TT). Meanwhile, the potential molecular mechanism was further explored by transcriptome sequencing (RNA-seq). The results indicated that TT could significantly restore heart RBCs intensity of thrombotic zebrafish, whilst decreasing RBCs accumulation in the caudal vein. The transcriptome analysis revealed that the preventive effect of TT on thrombosis could be mostly attributed to changes in lipid metabolism related signaling pathways, such as fatty acid metabolism, glycerollipid metabolism, ECM-receptor interaction and steroid biosynthesis signaling pathway. This study demonstrated that Tibetan tea could alleviate thrombosis by reducing oxidative stress levels and regulating lipid metabolism.

## Introduction

Ischemic cardiovascular disease is considered as the leading cause of morbidity and mortality worldwide. Among Ischemic cardiovascular diseases, thrombotic diseases cause necrotic tissues and further induce various serious cardiovascular and cerebrovascular diseases [1]. Thrombosis factors encompass a variety of factors, including abnormalities in platelet function, extrinsic and intrinsic coagulation factors, abnormal lipid metabolism, and alterations in blood flow [2]. Dyslipidemia, characterized by elevated levels of cholesterol, triglycerides, and low-density lipoprotein cholesterol (LDL-C), is closely associated with thrombosis [3]. These lipids tend to accumulate on the inner lining of blood vessels, leading to the development of atherosclerosis, which in turn slows down blood flow and promotes the formation of blood clots [4–6]. As such, managing blood lipid levels and preventing lipid deposition on the vascular wall represent effective strategies for reducing the risk of thrombotic events.

Tea is one of the most consumed beverages in the world [7]. There are six categories of tea leaves based on manufacturing process: unfermented green tea, lightly fermented yellow and white tea, semi-fermented oolong tea, fully fermented black tea, and post-fermented dark tea [8, 9]. Tibetan tea is a dark tea made through fixing, rolling, pile fermenting, autoclaving, and drying. The unique compounds in Tibetan tea are mainly formed during pile fermentation, which accelerates the aging of raw tea leaves [10]. The complex enzymatic action of microorganisms during pile fermentation have changed the compounds in the tea leaves, leading to the unique characteristics of Tibetan tea [11]. Tibetan tea is rich in tea polysaccharides, tea polyphenols (including catechins and their oxidized polymers), theaflavins, thearubigins and other bioactive components after pile fermentation [12–14]. Epidemiological studies showed that these active ingredients have the potential to prevent cardiovascular diseases and can reduce the LDL cholesterol and total cholesterol in obese patients [15–17]. Among them, tea polyphenols are found to increase the number of circulating angiogenic cells (CACs), thus protecting the blood vessels of hypertensive patients [18, 19]. Theaflavins and their gallates and isoamyl gallate are potent inhibitors of platelet aggregation and platelet activation [20]. In addition, catechins (GTC) were found in early studies to reduce platelet aggregation and thrombus formation in diabetic rats [20, 21]. Currently, there is a growing body of research examining the effects of green tea, white tea, and black tea on thrombosis and cardiovascular diseases [22–26]. However, there has been relatively little research on the effects of Tibetan tea in this regard.

In this study, we utilized arachidonic acid (AA) to develop a thrombus model in zebrafish, and evaluated the efficacy of Tibetan tea in alleviating thrombosis by measuring blood flow velocity, red blood cell aggregation, and oxidative stress levels. To gain further insight into the preventive mechanism of Tibetan tea in alleviating thrombosis, we employed RNA-seq and qPCR arrays. Our findings provide a valuable theoretical foundation for the use of Tibetan tea in the treatment of cardiovascular diseases.

## Materials and methods

### Chemicals and reagents

Arachidonic acid (AA, CAS 506-32-1) was purchased from Shanghai Yuanye Biotechnology Co, LTD. Aspirin was purchased from MedChemExpress. Tibetan tea was purchased from Sichuan Jixiang Tea Co., Ltd. anhydrous sodium acetate and DMSO were purchased from Shenggong Bioengineering (Shanghai) Co., Ltd. Anhydrous ethanol was purchased from Xilong Science Co., Ltd. SOD, MDA, ROS and other detection kits were purchased from Nanchang Excellence Biotechnology Co, LTD. TransStart Green qPCR SuperMix (AQ141-02) was purchased from Jiangxi Biyou Technology Co, LTD.

### Animal care ethics

Zebrafish was purchased from the China Zebrafish Resource Center. All experiments were executed following the Guide for the Committee on the Laboratory animal welfare and ethics committee of Gannan normal university(Protocol Number: gnnu2022-0628), after seeking proper approval.

### Zebrafish husbandry and embryo collection

Transgenic *Tg(gata1*:*DsRed)* zebrafish with enhanced expression of red fluorescent protein (DsRed) in the blood corpuscle were purchased from China Zebrafish Resource Center. According to institutional protocols of animal care, those zebrafish were kept in flow-through tanks with aerated freshwater at 28 ± 0.5°C under a 14/10-hour light/dark cycle and fed with freshly hatched brine shrimp.

To collect embryos, males and females were placed in mating tank at the ratio of 1:1 to 1:2 and separated by a barrier. The next morning, the barrier was removed, and the females started to lay eggs. The embryos were collected within half an hour. After removing dead and unfertilized eggs, feces, and other debris, the viable embryos were washed several times with egg water, and then incubated at 28.5°C for 24 h. Melanin production was inhibited by adding 1-phenyl-2-thiourea (PTU).

### Tibetan tea water extract

1.5 g of Tibetan tea was added to 80 mL of purified water, which was then put in a microwave oven and boiled for 6 mins and strain, then fixed the volume to 50 mL. Tibetan tea water extract was stored in a -20°C refrigerator for experiments [27].

### Zebrafish thrombosis modeling and drug treatment

In this study, AA was used to induce thrombus formation and aspirin, a clinically approved antithrombotic drug, was used as a positive control [28, 29]. Among them, aspirin is added to zebrafish 2 dpf (day post fertilization) for drug prevention, and AA is added to zebrafish 3 dpf for model establishment. It is noteworthy that thrombosis model was optimized to be under 30 μM concentration for 45 mins in our study, considering the age of zebrafish embryos and the reduction effect of the aspirin.

In order to further screen the optimal treatment concentration, According to the study of Li et al [30], we selected the following concentrations to evaluate the use dose of Tibetan tea 8 hours-post- fertilization (hpf) zebrafish embryos were exposed to 0.3 g/L, 0.9 g/L, 1.5 g/L, 2.1 g/L, 2.7 g/L, 3.3 g/L, 3.9 g/L, 4.5 g/L, 5.1 g/L, and 5.7 g/L of Tibetan tea for 24 hours to investigate the hatchability and developmental status. The medium was changed to fresh Tibetan tea after 12 hours.

This study included control group, AA (Arachidonic acid) group and TT (Tibetan tea) group. The control group was treated with DMSO, and the AA group was treated with 30 μM AA in zebrafish 3 days-post-fertilization (dpf) for 45 mins where AA was dissolved in the same concentration of DMSO as the control group. The TT group was treated with the optimal concentration of Tibetan tea water extract at 2 dpf for, and the same concentration of AA as the AA group was added for 45 mins. In all experiments, zebrafish were placed in six-well plates for treatments. In order not to affect zebrafish development, 20 embryos were placed in each well. Zebrafish were placed at 28.5°C in an incubator during all treatment.

### O-dianisidine staining

After AA treatment, the embryos from each group were stained with 0.6 mg/mL O-dianisidine for 20 mins in a dark environment, which was used to detect RBC level through quantitative imaging analysis.

### Analysis of blood flow and red blood cell (RBC) aggregation

In order to record blood flow videos, the embryos were fixed in 1% of low melting agarose without anesthesia. Blood flow velocity was calculated using DanioScope1. Blood flow of 20 zebrafish embryos with 3 biological replicates was analyzed in each group. RBC aggregation of Transgenic zebrafish *Tg(gata1*:*DsRed)* was also analyzed. The embryos were fixed in 1% of low melting agarose with 0.4% tricaine anesthesia before microscopy imaging. Images were taken under a Leica M205 FA stereoscopic microscope (Germany). The blood flow videos can be found in the S1 Video. The effect rate of thrombus prevention in the treatment groups was calculated by the number of zebrafish with reduced thrombus over the number of successful thrombus models.

### Oxidative stress analysis

DCFH-DA, a fluorescent indicator of H2O2 or other ROS, was used as a marker for cellular oxidative stress. The embryos were incubated in 1000x diluted DCFH-DA at 28.5°C for 30 mins in the dark and imaged under a fluorescence microscope (Leica 322 M205 FA stereo microscope, Germany). Fluorescence intensity of ROS staining was calculated using Image J (NIH, USA). SOD and MDA levels were measured using suitable kits according to the kit instructions. Total protein level was quantified using Coomassie Brilliant Blue staining. Absorbance was measured using a multifunctional micrometer (PerkinElmer Victor nivo, USA) with each sample measured three times.

### RNA isolation to construct library

30 embryos were collected from each group at 3 dpf and washed five times with phosphoric acid buffer. RNA was extracted and quantified using Trizol reagent (Invitrogen, Carlsbad, USA). After determining the RNA quality and quantity, the libraries were sequenced on an Illumina HiSeq X Ten platform. Then, 150 bp paired-end reads were obtained.

### RNA-seq analyses

Transcript assembly and functional assignment were performed as described earlier. Differential expression analyses were performed between groups with duplicates using the DESeq2 R package (1.16.1). Gene Ontology (GO) analysis of differentially expressed genes (DEGs) was performed using the clusterProfiler R package with gene length bias corrected. GO terms with corrected P value <0.05 were considered significantly enriched by DEGs. Statistical enrichment of DEGs in KEGG pathways was evaluated in clusterProfiler R package.

### RNA extraction and quantitative real-time PCR

50 embryos were collected from each treatment group and washed five times with phosphoric acid buffer. The embryos were homogenized using the TriZol reagent (Invitrogen) to extract the RNA, which was then reverse-transcribed to cDNA using a Prime Script® RT reagent kit. Real-time PCR was performed using the Applied Biosystems Step-One-plus real-time PCR system (Analytic Jena, Germany /qTower 3G) with the SYBR Green detection kit and β-actin as the internal control. The primer sequences were shown in S1 Table. Each sample was tested in triplicates.

### Statistical analyses

Statistical analyses including one-way ANOVA and student t-test were performed using GraphPad Prism 5.0 Software. All values were shown as mean ± standard deviation. The resulted P values were annotated as follows: * for P < 0.05, ** for P < 0.01, and *** for P < 0.001 unless otherwise noted.

## Results

### Chemical composition of Tibetan tea

Tibetan tea is a type of dark tea that exhibits various health-promoting benefits, mainly attributed to its multiple active ingredients. Therefore, a preliminary assessment of the chemical composition of Tibetan tea has been carried out. Its total polyphenol, total flavonoid and total polysaccharide contents were 61.3 mg/g, 80.7 mg/g and 66.3 mg/g, respectively. The results of previous studies proved that the total tea polyphenol content of the three Tibetan teas ranged from 56.82–101.66 mg/g. Our findings were similar to those described above.

### Validation of thrombus model in zebrafish

**Fig 1:**
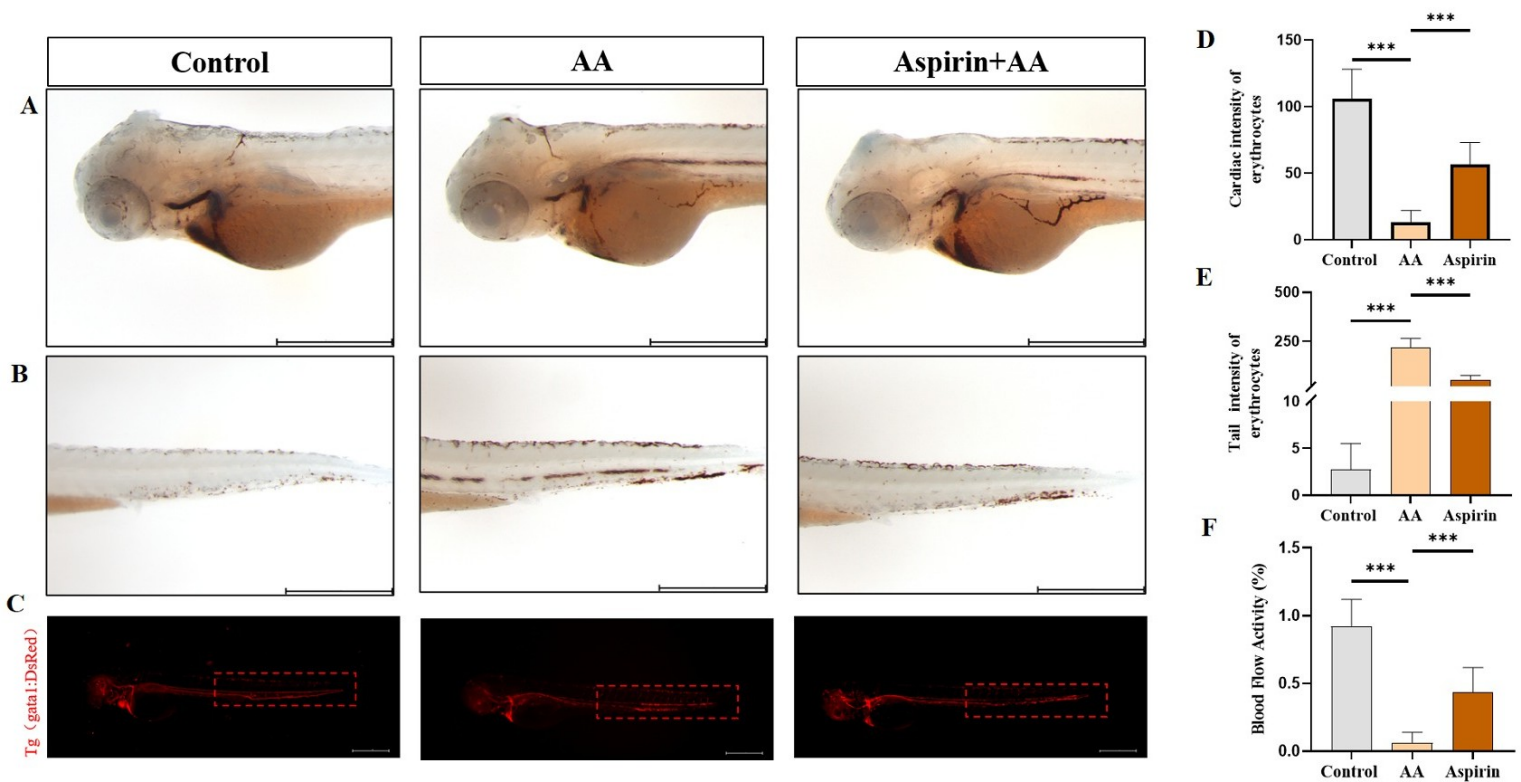
The establishment of zebrafish thrombus model. Representative images (A) and quantification (D) of o-dianisidine staining at the heart regions of control, AA and aspirin groups. Representative images (B) and quantification (E) of o-dianisidine staining at the tail regions control, AA and aspirin, treated. (C) Blood cell aggregation of Tg(gata1:DsRed) zebrafish in control group, AA group, and aspirin group. (F) Blood flow velocity frequency diagram of zebrafish in control, AA, and aspirin. Compared with the control group; compared with the model group,* for P < 0.05, ** for P < 0.01, *** for P < 0.001.(Note: The length of the scale bar in the figure is 500 μm).

After AA treatment and o-benzidine staining, 3 dpf zebrafish showed significantly reduced number of RBCs in the heart and significantly increased number of RBCs in the tail(P<0.001). Aspirin treatment, however, was able to mitigate the change caused by AA treatment(P<0.001) (Fig 1A, 1B, 1D and 1E). The fluorescence microscope observation showed consistent result as staining, confirming robustness of the zebrafish thrombosis model (Fig 1C).

### Selection of the doses administered in the TT group

**Fig 2:**
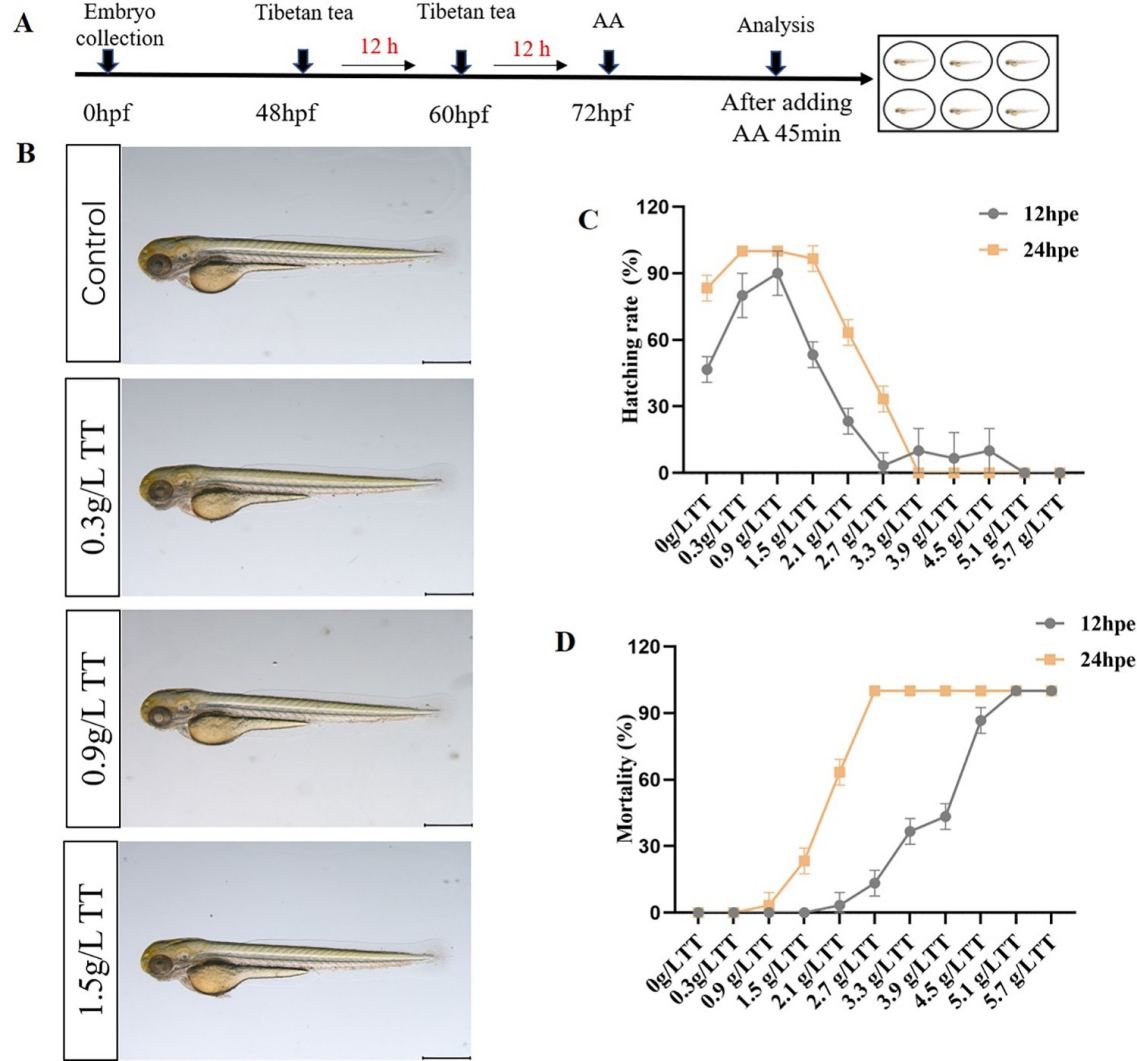
Screening of optimal treatment concentration of Tibetan tea. Illustration of the timeline of drug protection and AA treatment. (B) Phenotype diagram of the effect of different concentrations of TT on zebrafish. (C) Effects of different TT concentrations on the hatching rate of zebrafish. (D) Effects of Different TT Concentrations on Zebrafish Mortality. (Note: The length of the scale bar in the figure is 500 μm).

We investigated the effect of Tibetan tea on zebrafish embryos. It was found that at 24 hours after treatment, Tibetan tea of a higher than 1.5 g/L concentration significantly (P<0.001) increased the zebrafish mortality and had an impact on embryo malformation rate and hatchability. Tibetan tea in the range of 0.3 g/L-1.5 g/L had no obvious impact on the development of zebrafish embryos (Fig 2B–2D). As a result, 0.9 g/L Tibetan tea was used for the subsequent experiments.

### TT alleviates thrombus

**Fig 3:**
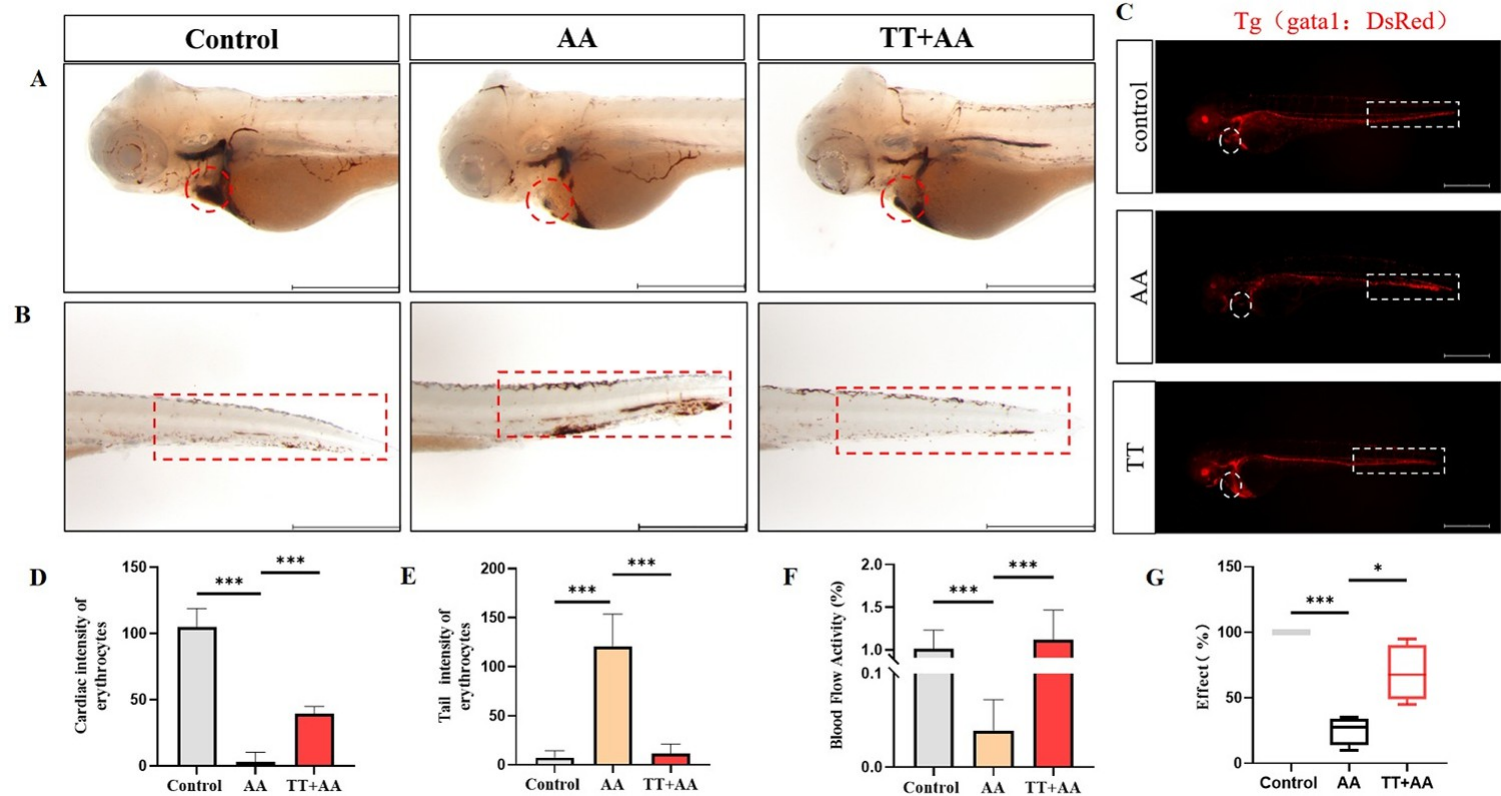
Effects of TT on thrombus model of zebrafish. Representative images (A) and quantification (D) of o-dianisidine staining at the heart regions of control, AA and TT groups. The heart regions are circled by red dotted line. Representative images (B) and quantification (E) of o-dianisidine staining at the tail regions control, AA,and TT treated. (C) Blood cell aggregation of Tg(gata1:DsRed) zebrafish in control group, AA group, and TT group. (F) Blood flow velocity frequency diagram of zebrafish in control, AA, and TT. (G) Effective rate of Tibetan tea rescue (%). Compared with the control group; compared with the model group,* for P < 0.05, ** for P < 0.01, *** for P < 0.001. (Note: The length of the scale bar in the figure is 500 μm).

The AA-induced thrombosis model was then used to study the effect of Tibetan tea on thrombosis. The results of o-benzidine staining showed that compared with the control group, the RBC level in the heart in the AA model group were significantly reduced (p<0.001), and the red blood cells in the tail were significantly increased (p<0.001). The RBC level in the heart in TT group was significantly increased (p<0.001) and that in the tail was significantly increased (p<0.001) compared with the AA group (Fig 3A, 3B, 3D, 3E). Blood flow statistics showed that Tibetan tea alleviated the slowed blood flow and blockage caused by AA (Fig 3F). The aggregation of RBCs in the heart and tail of zebrafish was confirmed through fluorescence microscopy (Fig 3C). In addition, fluorescence microscopy indicated that the blood circulation rate of the AA group was 10%-35% while that of the TT group was 65–95% compared to the control group (Fig 3G).

### Oxidative stress alleviation

**Fig 4:**
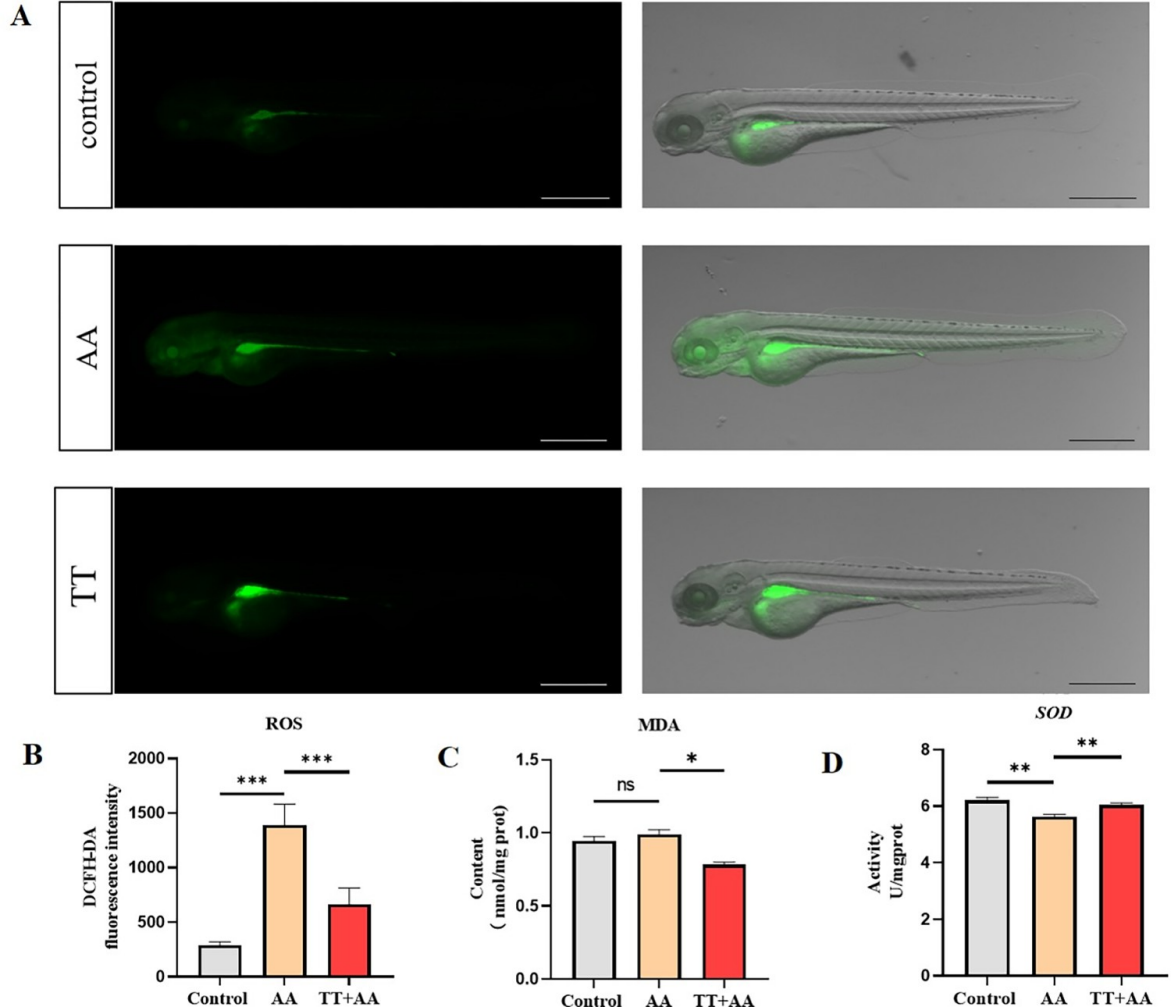
Effects of TT on oxidative stress levels in zebrafish thrombosis models. Representative images (A) and quantification (B) of the fluorescence signals of DCFH-DA staining in different treatment groups. Concentration of MDA (C) and SOD (D) in zebrafish in different treatment groups. Compared with the control group; compared with the model group,*P < 0.05, **P < 0.01, ***P < 0.001. (Note: The length of the scale bar in the figure is 500 μm).

The results of DCFH-DA staining showed that after AA treatment, the ROS fluorescence intensity of zebrafish was significantly increased (p < 0.001), while TT treatment significantly mitigated the trend (p < 0.001) (Fig 4A and 4B). We further analyzed MDA and SOD levels. The MDA level was increased in the AA group (p>0.05), while TT treatment significantly reduced it (p<0.05) (Fig 4C). The SOD level in the AA group was significantly decreased (p<0.01), while TT treatment significantly increased it back to normal (p < 0.01) (Fig 4D). The results suggested that Tibetan tea has antioxidant effects.

### RNA-seq analysis

**Fig 5:**
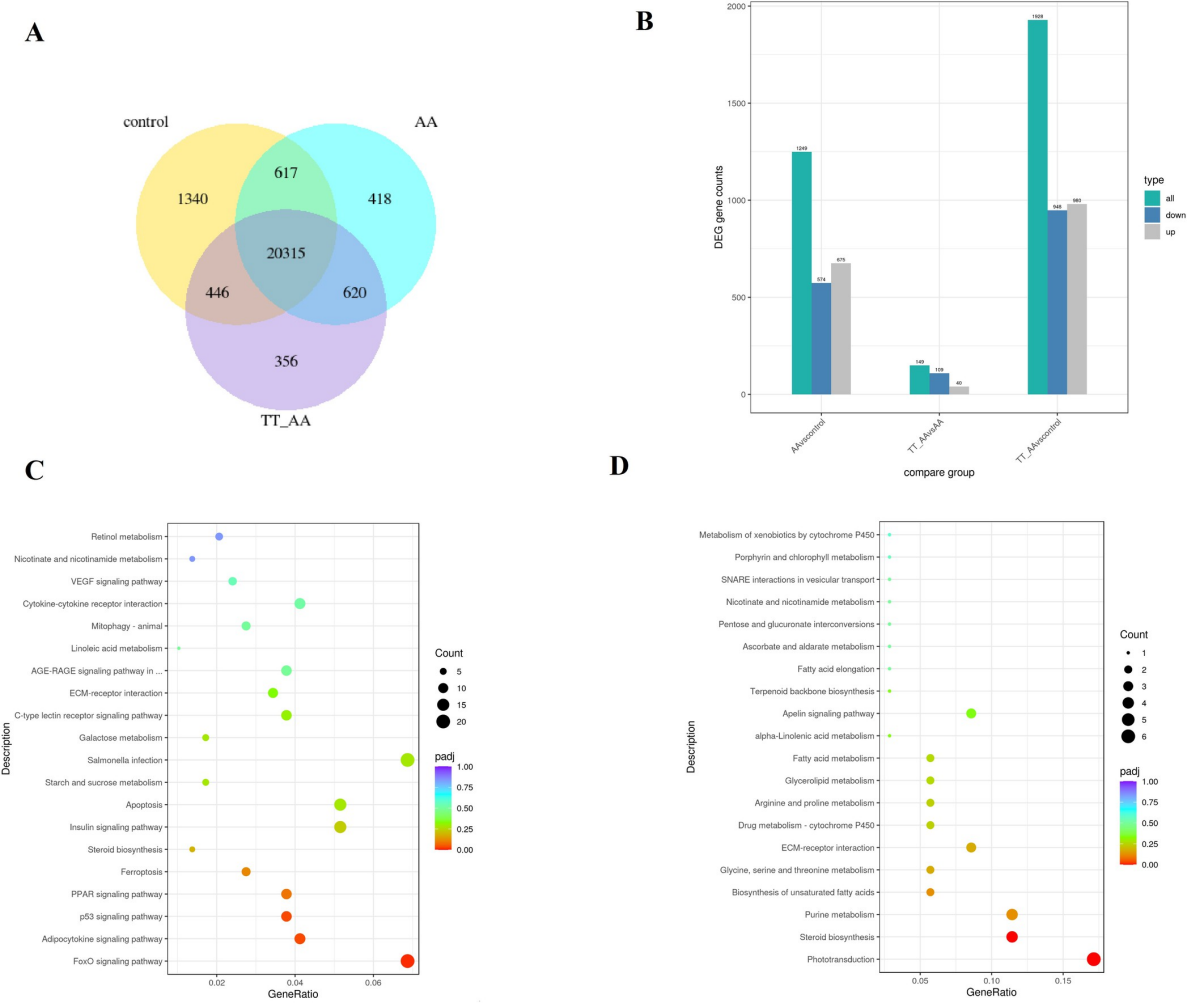
RNA-seq data analysis. (A) Venn diagram of gene counts expressed. (B) Significant DEG counts. (C) The KEGG pathway enrichment of DEGs between AA and control. (D) The KEGG pathway enrichment of DEGs between AA and AA _TT.

To reveal the underpinning mechanisms of Tibetan tea on thrombosis, we performed transcriptome analysis of zebrafish samples using RNA-Seq (S2 Table). The raw sequence data reported in this paper have been deposited in the Genome Sequence Archive (Genomics, Proteomics & Bioinformatics 2017) in National Genomics Data Center (Nucleic Acids Res 2021), China National Center for Bioinformation/ Beijing Institute of Genomics, Chinese Academy of Sciences, under accession number CRA008241 that are publicly accessible at https://bigd.big.ac.cn/gsa. The genes with a reads per kilobases per million (RPKM) ratios greater than twofold were defined as differentially expressed genes (DEGs). We used DEGseq to identify significant DEGs, including up- or down-regulated genes. Compared with the control group, 1249 DEGs were identified in the AA group, including 675 up-regulated genes and 574 down-regulated genes. Compared with the AA group, 149 DEGs were identified in the TT group, including 40 up-regulated genes and 109 down-regulated genes (Fig 5A and 5B).

We performed KEGG pathway analysis on each group of samples. The results showed that the DEGs between the AA and control groups were enriched in Adipocytokine signaling pathway, FoxO signaling pathway, ECM-receptor interaction, Linoleic acid metabolism and other signaling pathways. The DEGs between the TT and AA groups were enriched in fatty acid metabolism, glycerolipid metabolism, ECM-receptor interaction, steroid biosynthesis, biosynthesis of fatty acids, fatty acid ellongation and apelin signaling pathway (Fig 5C and 5D). It is worth noting that most of these pathways are related to lipid metabolism and cell adhesion.

### qRT-PCR

**Fig 6:**
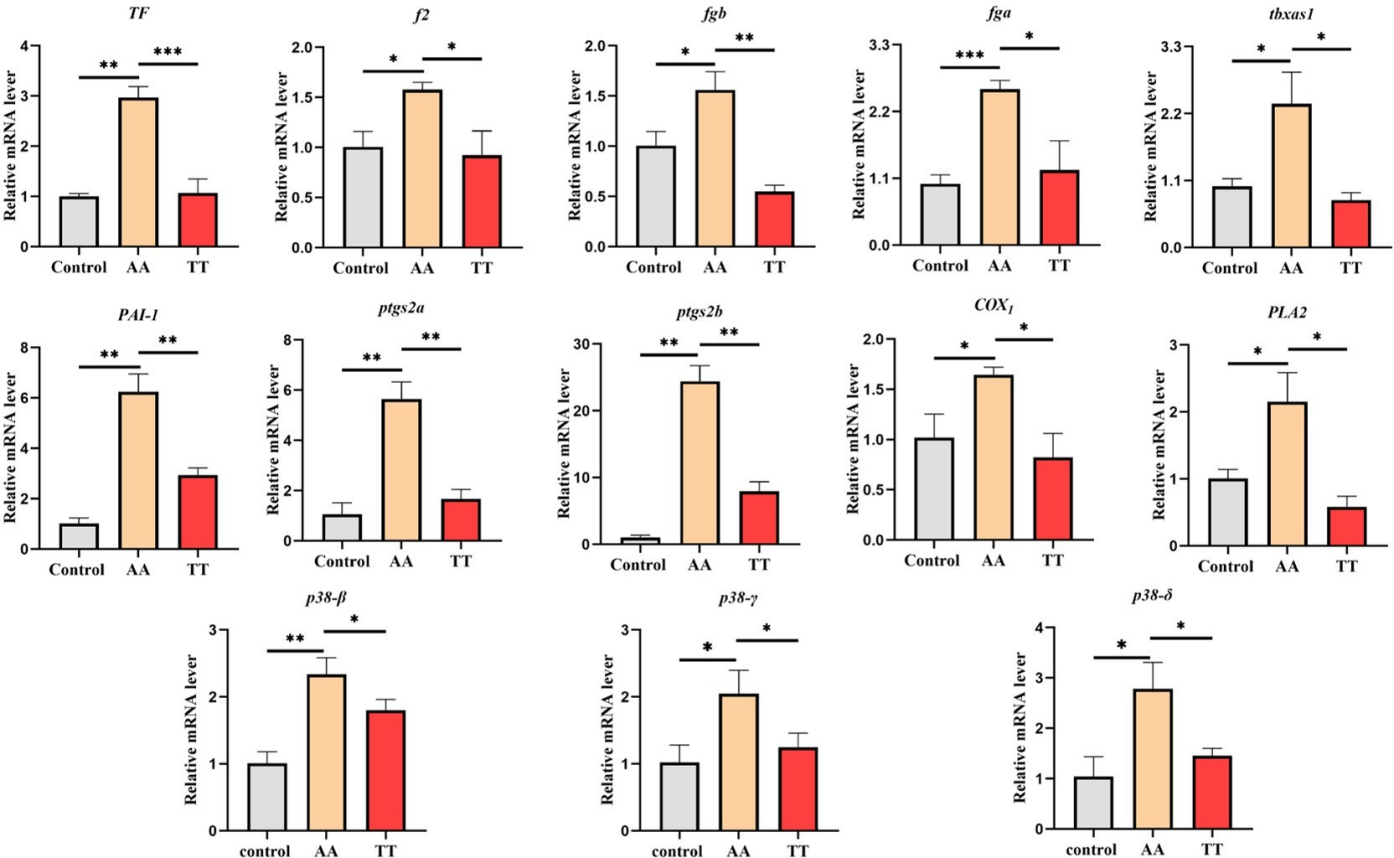
Changes of thrombus-related gene expression. Compared with the control group; compared with the model group,*P < 0.05, **P < 0.01, ***P < 0.001.

We identified the changes in the expression levels of thrombosis-related factors using qPCR, which include tissue factor (TF), coagulation factor (f2), fibrinogen α, β chains (fga, fgb), thromboxane synthase (tbxas1), prostaglandin peroxidase (ptgs2a, ptgs2b), plasminogen activator inhibitor (PAI-1), prostaglandin peroxidase (cox1), PLA2, p38β (MAPK11), p38γ (MAPK12), and p38δ (MAPK13). Compared with the control group, the transcription levels of all the above genes in the AA group were significantly up regulated, which were significantly alleviated after treatment with Tibetan tea (Fig 6).

## Discussion

Thrombosis is a pathological process in which fibrin and platelets aggregate to cause blood vessel blockage. It will obstruct the blood flow of the circulatory system and may lead to a series of thrombotic diseases including myocardial infarction, cerebral infarction, and atherosclerosis [31]. Arachidonic acid (AA) is a popular platelet agonist [32], a precursor for the biosynthesis of prostaglandins (PG), which has important effects on the blood and cardiovascular system. It can induce platelets to release endogenous ADP and activate platelet aggregation. AA can be converted to thromboxane A2 (TXA2), prostaglandins and leukotriglycerides through cyclooxygenase and lipoxygenase. TXA2 can constrict blood vessels, enhance platelet aggregation, and increase blood viscosity, while prostaglandin can expand blood vessels and inhibit platelet aggregation, both of which regulate platelet aggregation and vasoconstriction and maintains blood circulation [33, 34]. Aspirin is the most commonly used drug in the clinical treatment of cardiovascular and cerebrovascular diseases. It can inhibit the activation of platelets and relieve platelet aggregation by inhibiting cyclooxygenase and thus the synthesis of thromboxane and prostacyclin [35]. zebrafish has the same factors of platelet adhesion, activation, aggregation, and release as human. It also has coagulation factors and platelet receptors. It responds to common anticoagulant and antiplatelet drugs in clinical practice and is a novel animal model to study coagulation and thrombosis in mammals [32, 36]. Therefore, we constructed a zebrafish thrombus model through AA induction, and found that Tibetan tea can alleviate the reduction of red blood cells in the heart and the aggregation of red blood cells in the tail during thrombus formation. At the same time, we monitored the blood flow rate of zebrafish, and the results showed that Tibetan tea can improve the slow blood flow rate in the process of thrombus formation. The above results show that Tibetan tea has the effect of alleviating thrombosis.

To further elucidate the mechanism by which Tibetan tea alleviates thrombosis, we conducted transcriptomic analysis on zebrafish embryos in each experimental group. KEGG pathway analysis revealed that Tibetan tea exerts its thrombus-alleviating effects primarily through the regulation of key signaling pathways, including fatty acid metabolism, glycerolipid metabolism, ECM-receptor interaction, steroid biosynthesis, biosynthesis of fatty acids, fatty acid elongation, and apelin. Notably, these pathways are predominantly linked with lipid metabolism. Lipids are a family of biomolecules with important structural and signaling functions in platelets. Arachidonic acid (AA) is a polyunsaturated fatty acid that is present in high concentrations in platelets. Specifically, up to 25% of the phospholipid fatty acids found in platelets are AA. This fatty acid plays a critical role in platelet function and has been implicated in the pathogenesis of several cardiovascular diseases, including thrombosis and atherosclerosis [5, 37]. Their formation and metabolism are controlled by enzymes and signal transduction pathways and dysregulation can lead to significant defects in platelet function and disease [38]. Abnormal blood lipid is a pre-thrombotic phenotype, a disorder characterized by high levels of circulating lipids such as low- density lipoprotein (LDL), total cholesterol (TC) or triglycerides and/or decreased levels of high- density lipoprotein (HDL) [5]. Lipid metabolism and the coagulation cascade are interrelated, and certain lipoproteins involved in lipid metabolism may influence the coagulation response by acting as either procoagulants or anticoagulants [39, 40]. We detected the expression of key coagulation factors in the clotting cascade by qPCR. The results showed that treatment with AA significantly increased the expression of factors related to the zebrafish clotting cascade, including TF, f2, fga, fgb, tbxas1, and PAI-1, while treatment with Tibetan tea significantly decreased the expression of these genes.

Meanwhile, dyslipidemia can cause oxidative stress, thereby affecting the occurrence and development of thrombosis. Oxidation of low-density lipoproteins (OxLDL) results in the release of oxidized cholesterol and phospholipids among other fatty acid products. OxLDL can alter the coagulation cascade in two independent ways. The first form is via inhibiting plasma proteins of the intrinsic pathway FVIII, FIX and FXI and by stimulating the production of TF by endothelial cells [41]. The second mechanism involves the interaction between the platelet surface protein CD36 and oxidized low-density lipoprotein (oxLDL), which can promote platelet activation and contribute to thrombosis [42]. p38 MAPK is an ROS-sensitive signaling pathway, and an increase in ROS can promote endothelial cell apoptosis and activate inflammation, leading to thrombosis [43]. Reactive oxygen species (ROS) enhance p38MAPK signaling to induce A2 phospholipase activation through phosphorylation [44]. PLA2 can releasing AA into the cytoplasm, and cox1 converts AA to prostaglandin G2 (PGG2), an intermediate in the synthesis of TxA2 [45]. Therefore, we evaluated the antioxidant effect of Tibetan tea by detecting ROS, malondialdehyde (MDA) and superoxide dismutase (SOD) activity in zebrafish. Using qPCR, we also verified that oxidative stress-related genes (ptgs2a, ptgs2b, cox1, PLA2 and p38β, p38γ, p38δ) were significantly up-regulated after AA treatment. The results indicated that AA could induce thrombosis by increasing oxidative stress, and Tibetan tea could significantly reduce oxidative stress.

Distinct chemical components present in various types of tea can modulate specific lipid metabolism-related proteins [46]. Tea polysaccharides and polyphenols have demonstrated efficacy in inhibiting body fat accumulation and improving blood lipid levels in mice. The mechanism underlying their anti-obesity effects primarily involves the modulation of various lipid metabolism-related pathways, such as fatty acid biosynthesis, sterol hormone biosynthesis, unsaturated fatty acid biosynthesis, fatty acid elongation, glycerol-lipid metabolism, and glycerophospholipid metabolism [47, 48]. Tea polyphenols are the main constituents of Tibetan tea. During the fermentation process, these polyphenols undergo oxidation by polyphenol oxidase, resulting in the production of oxidized tea polyphenols. It has been found that these oxidized tea polyphenols can effectively reduce the accumulation of lipids in rat liver adipose tissue and promote lipid metabolism in vivo [49]. Numerous studies have demonstrated that Tibetan tea contains various bioactive compounds with potent antioxidant effects [12, 13]. In summary, Tibetan tea may achieve the effect of alleviating blood clots by regulating lipid metabolism and anti-oxidation through a variety of active ingredients.

## Conclusion

In the study we found that Tibetan tea can reduce AA-induced thrombosis and oxidative stress in zebrafish. RNA-seq and qPCR analysis identified that the effects of Tibetan tea on thrombosis were attributed to the regulation of lipid metabolism and cell adhesion. Our findings provided important insights into the molecular mechanisms by which Tibetan tea prevents and reduces thrombosis. Further research may be needed to identify specific monomeric components in Tibetan tea that contribute to beneficial effects.

## Supporting information

S1 Table Primers sequences for real-time quantitative PCR. (DOCX)

S2 Table Summary of RNA-sequencing data. (DOCX)

S1 Video Zebrafish blood flow videos. (ZIP)
