# Supplementary material for: Preventive effect and mechanism of Tibetan tea extract on thrombosis in arachidonic acid-induced zebrafish determined via RNA-seq transcriptome profiles
Source: PLoS One. 2023 May 19;18(5):e0285216. doi: 10.1371/journal.pone.0285216 (PMC10198549; doi:10.1371/journal.pone.0285216)
Supplement: S1 Table — (DOCX) [file pone.0285216.s001.docx]

**Supplementary Table 1 Primers sequences for Real-Time Quantitative PCR**

| Gene | Forward Sequence | Reverse Sequence |
| --- | --- | --- |
| TF | CTGACGTCCTGTCTGAACCC | GGTCTACCAATGTCCGTGTCA |
| f2 | TGGAAGGAAAACCTGAACCG | TGAGGTCCAAGACTCCCGAA |
| fga | CTACGCACATGCAAAGGCTC | TTTGCCCGTTCCGCTCTTAT |
| fgb | CTCAGAGAGCCAAGTGCCAA | CCAACGCCTGCCAAAATCAA |
| tbxas1 | TTTGATGTGGTCAACGACGC | TGCTTGTCTCATAGCCAGCC |
| PAI-1 | GAGCGTCCCACACCAGATAG | GCACTCCAGATGGGAGGAAC |
| ptgs2a | CTGCACTACCCCTGAGCTTC | TCAGCATTGTACGTCGGTGG |
| ptgs2b | CAACGCCATCTTTGGGGAGA | CATAGGACATGGCCCGTTGA |
| cox1 | AAGTACCAGGTGCTCAACGG | AAGGTAGCGTACATGCCGAG |
| PLA2 | CCGACACACTCCCTGAACAA | TGTGTATGTTGGGACGACGG |
| p38β | TCTGTCGGATGCATCATGGG | TGTGCGGAAGAGACTGGATG |
| p38γ | GGCAGAGATGCTGCTAGGAA | CAGATGCCGTCTAACACTTT |
| p38δ | GTGCGTTGGCACACAGTTAT | TCTGCATCAGACCCACTATCAC |
| *β*-actin | CGAGCAGGAGATGGGAACC | CAACGGAAACGCTCATTGC |
