# Supplementary material for: Preventive effect and mechanism of Tibetan tea extract on thrombosis in arachidonic acid-induced zebrafish determined via RNA-seq transcriptome profiles
Source: PLoS One. 2023 May 19;18(5):e0285216. doi: 10.1371/journal.pone.0285216 (PMC10198549; doi:10.1371/journal.pone.0285216)
Supplement: S2 Table — (DOCX) [file pone.0285216.s002.docx]

**Supplementary Table 2 Summary of RNA-sequencing data**

| sample | raw_reads | raw_bases | clean_reads | clean_bases | error_rate | Q20 | Q30 | GC_pct |
| --- | --- | --- | --- | --- | --- | --- | --- | --- |
| C_1 | 44731994 | 6.71G | 44009432 | 6.6G | 0.03 | 96.33 | 90.41 | 42.47 |
| C_2 | 44985110 | 6.75G | 44056830 | 6.61G | 0.03 | 96.75 | 91.26 | 40.97 |
| C_3 | 43737584 | 6.56G | 43074442 | 6.46G | 0.03 | 97.58 | 93.15 | 44.14 |
| AA_1 | 45640470 | 6.85G | 44814338 | 6.72G | 0.03 | 97.29 | 92.62 | 46.28 |
| AA_2 | 45383902 | 6.81G | 41795010 | 6.27G | 0.03 | 96.76 | 91.35 | 43.33 |
| AA_3 | 44684964 | 6.7G | 41883952 | 6.28G | 0.03 | 97.04 | 92.06 | 44.08 |
| TT_AA_1 | 46754856 | 7.01G | 45897230 | 6.88G | 0.03 | 97.08 | 92.14 | 46.21 |
| TT_AA_2 | 41840260 | 6.28G | 38547362 | 5.78G | 0.03 | 97.45 | 92.84 | 45.97 |
| TT_AA_3 | 44400980 | 6.66G | 42489026 | 6.37G | 0.03 | 96.66 | 91.19 | 45.7 |

Note: raw_ Reads: the number of reads in the raw data, clean_ Reads: the number of filtered reads of the raw data, clean_ Bases: base number of filtered raw data (clean base = clean reads * 150bp), error_ Rate: overall sequencing error rate of data, Q20: percentage of bases with phred value greater than 20 in total bases, Q30: percentage of bases with phred value greater than 30 in total bases, GC_ PCT: percentage of G and C in four bases of clean reads.
